# Papillary Thyroid Carcinoma: Ectopic Malignancy versus Metastatic Disease

**DOI:** 10.1155/2017/9707031

**Published:** 2017-06-18

**Authors:** Yanery's Agosto-Vargas, Madeleine Gutiérrez, José Hernán Martínez, Michelle Mangual-Garcia, Coromoto Palermo, Sharon Vélez-Maymi, Luis Hernández-Vázquez, Samayra Miranda-Rodríguez, Alex González-Bossolo, Ernesto Solá-Sánchez, Marianne Hernández-Negrón

**Affiliations:** Endocrinology, SJCH, San Juan, PR, USA

## Abstract

Papillary thyroid carcinoma frequently metastasizes to regional lymph nodes. However, cervical lymph node metastasis as a sole manifestation of occult papillary thyroid carcinoma is rarely observed. Ectopic thyroid is an uncommon condition defined as the presence of thyroid tissue at a site other than pretracheal area. Approximately 1–3% of all ectopic thyroid tissue is located in the lateral neck. This entity may represent the only functional thyroid tissue in the body. Malignant transformation of ectopic thyroid is uncommon; but even rarer is the development of papillary carcinoma on it. We present a case of a 33-year-old man with an incidental lateral neck mass diagnosed after a motor vehicle accident. Total thyroidectomy and lymph node resection were completed without evidence of papillary thyroid carcinoma. Malignant transformation of heterotopic thyroid tissue was the final diagnosis. The possibility of an ectopic thyroid cancer should be considered in the differential diagnosis of a pathological mass in the neck. The uniqueness of this case strives in the rarity that the thyroid gland was free of malignancy, despite ectopic tissue being positive for thyroid carcinoma. Management strategies, including performance of total thyroidectomy, neck dissection, and treatment with radioiodine, should be based on individualized risk assessment.

## 1. Background

Ectopic thyroid gland is defined as thyroid tissue localized outside of the second to fourth tracheal cartilages [1]. In rare cases, aberrant migration can result in lateral ectopic thyroid tissue [2]. Approximately 1–3% of all ectopic thyroid tissue is located in the lateral neck [2]. Its incidence is about 1 per 300,000 cases, being most common in females [3]. However, incidence may be underestimated, since ectopic tissue is often found incidentally [4]. Carcinoma arising from ectopic thyroid tissue is quite rare, comprising less than 1% of all thyroid carcinoma cases [3], with only a few reported in the literature. Papillary thyroid carcinoma is the most frequently identified subtype of carcinoma arising from ectopic tissue [4]. We present a case of a 33-year-old man with lateral neck papillary carcinoma without evidence of a thyroid gland lesion.

## 2. Case Presentation

**Figure 1:**
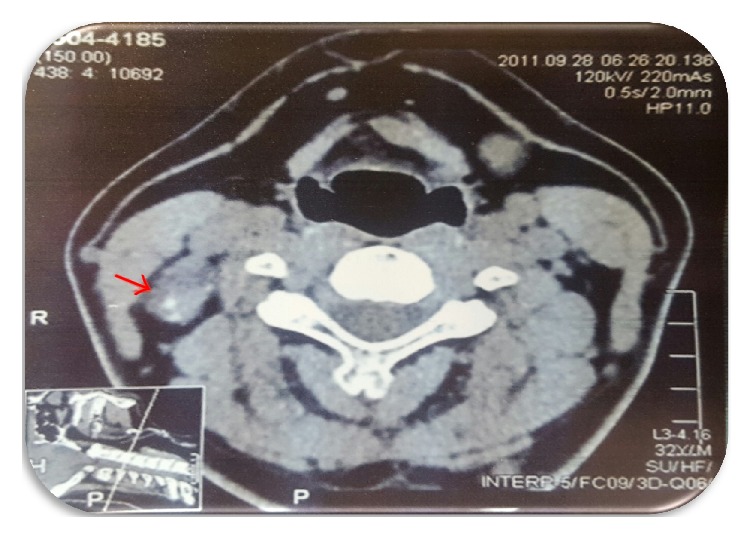
Cervical spine CT: complex right neck 2.6 cm × 2.3 cm mass incidentally noted as the posterior aspect of the jugular chain. It showed predominant decrease (20 HU) with a solid nodule (40 HU) posteriorly containing punctuate calcifications measuring 1.3 cm. Red arrow indicates right neck mass.

**Figure 2:**
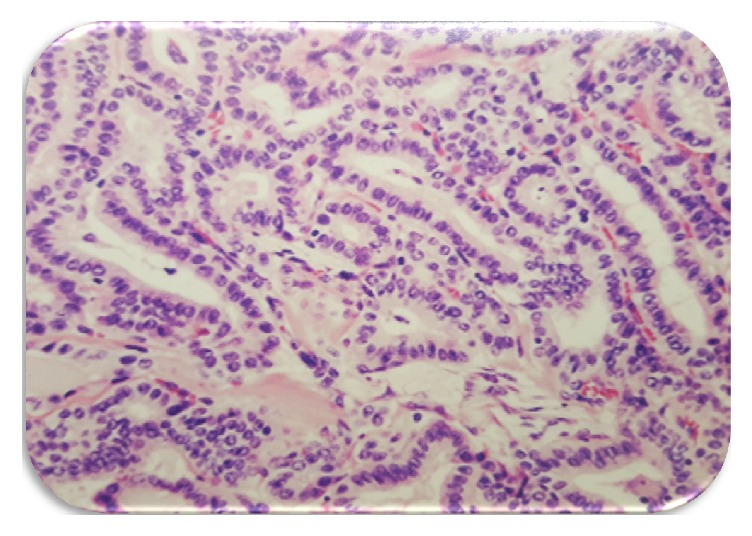
Excisional lymph node biopsy. Metastatic well-differentiated papillary thyroid carcinoma in lymph node.

**Figure 3:**
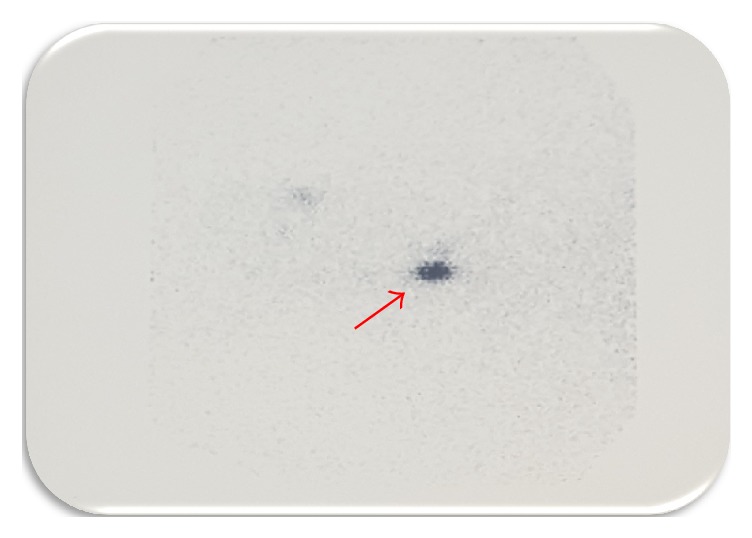
Whole-body scan. Functional thyroid tissue remnants in the thyroid bed with multiple enlarged neck lymph nodes. Red arrow indicates functional thyroid tissue remnants in the thyroid bed.

This is the case of a 33-year-old man without significant medical history who was incidentally diagnosed with a right neck cystic mass by computer tomography (CT) after a motor vehicle accident (Figure 1). Patient denied diaphoresis, palpitations, diarrhea, constipation, mood changes, or any other symptoms. Physical exam revealed a right-sided tender neck mass, without any other remarkable findings. He did not have history of neck irradiation, thyroid disease, or family history of thyroid cancer. Thyroid function tests were within normal limits (TSH: 1.5  IU/mL). Excisional biopsy of the neck mass reported metastatic, well-differentiated, thyroid papillary carcinoma within lymph node tissue. Pathologic description consisted of a nodular segment, tannish, rubbery specimen with attached membranous cystic tissue. The pathological specimen (lymph node) was distorted and had a well-defined cystic structure within it. While the cystic structure measured 2 cm × 1.5 cm × 1 cm, the lymph node measured 1.5 cm × 1 cm (Figure 2). Due to previous findings, he underwent total thyroidectomy with right neck dissection in order to rule out occult primary carcinoma of the thyroid. Histopathological findings revealed a normal thyroid gland without evidence of papillary thyroid carcinoma and sixteen right neck lymph nodes without evidence of metastasis. Thyroid pathology was meticulously reviewed, without any evidence of papillary thyroid carcinoma identified. After surgery, thyroid hormone replacement was started. One month after procedure, thyroglobulin was 133.61 ng/ml (1.15–130.77 ng/ml) and thyroglobulin antibodies were 11.8 uU/ml (negative, less than 45 uU/ml). Thyroid scintigraphy reported functional thyroid remnants at the right thyroid bed. Ultrasonography evaluation revealed hypoechoic foci within the thyroid beds bilaterally, likely secondary to postsurgical granulation tissue versus residual thyroid tissue. A right, level 2A lymph node seen measured 2.1 × 1 cm with loss of normal lymph node morphology, without microcalcifications or internal increase in vascularity. Another lymph node at level 3 measured 2 cm × 0.7 cm × 8.7 cm, without worrisome features. Fine needle aspiration biopsy of the aforementioned nodules showed papillary thyroid carcinoma. Final diagnosis was malignant transformation of heterotopic thyroid tissue. Whole-body scan showed functional thyroid tissue remnants in the thyroid bed with multiple enlarged neck lymph nodes (Figure 3). At that time, TSH was elevated (44.3 IU/mL) and free T4 was suppressed (0.58 ng/dl; normal value: 0.75–1.54 ng/dl). The patient was referred to nuclear medicine for radioiodine therapy. Radioiodine ablation 142.2 mCi was given. After appropriate cessation of hormone replacement therapy, whole-body scan showed no nodules uptake.

## 3. Discussion

The thyroid gland is derived from two anlages during the embryologic development: a large median endodermal anlage and two lateral anlages [1]. The median anlage produces most of the thyroid parenchyma, while the lateral anlage is derived from the fourth pharyngeal pouch and contributes 1% to 30% of the thyroid weight [1]. While the ectopic tissue more commonly develops secondary to abnormal cellular migration during the embryological development [5], it may also arise as a consequence of anomalous tissue implantation during surgical procedures on a normally located thyroid gland and metastasis of thyroid carcinoma [6]. The ectopic tissue is more commonly found along the cervical midline, with said position accounting for roughly 90% [6] of the cases. The remaining 10% of cases are distributed amongst the anterior tongue, larynx, trachea, esophagus, mediastinum, pericardium, diaphragm, and, rarely, brachial cyst [4]. Primary malignant transformation of ectopic thyroid tissue is an exceedingly uncommon event; only 43 cases have been reported in the literature. From these cases, ten were identified as papillary thyroid carcinomas [6]. Precise epidemiological data regarding the incidence and prevalence of lateral neck ectopic thyroid carcinomas are lacking, mainly due to the difficulty in distinguishing between metastatic diseases related to a thyroid primary carcinoma and true primary malignancy of the ectopic tissue [7]. Therefore, pathological analysis of tissue samples is paramount to accurately identify primary malignancies of ectopic thyroid tissue, once the clinical suspicion is established [7]. Our case presents an uncommon presentation by the fact that primary thyroid malignant lesion was located at the lateral ectopic tissue without evidence of active disease in the thyroid gland.

Due to the rarity of such cases, there are no standard, evidence-based guidelines regarding the optimal treatment of primary ectopic lateral neck thyroid carcinoma. In terms of work-up, literature suggests fine needle aspiration of identified neck masses as the test of choice to establish diagnosis. Several treatment options have been attempted and reported in different case reports. Total thyroidectomy with excision of the ectopic thyroid tissue and bilateral neck dissection was proposed by Wang et al. for a patient with submental ectopic papillary thyroid carcinoma presenting as bilateral progressively growing neck masses [8]. Total thyroidectomy with ipsilateral modified neck dissection followed by radioactive iodine therapy has also been reported to grant a favorable prognosis [9]. As previously mentioned, our patient was treated with total thyroidectomy followed by radiation therapy. Postradioiodine whole-body scan had no evidence of residual disease. Unfortunately, the patient was lost to follow-up and further posttreatment studies could not be completed. The purpose of this case is to raise awareness about the possibility to have thyroid carcinoma arising from ectopic tissues without evidence of malignancy in the thyroid gland. To our knowledge, there are few cases in literature where ectopic thyroid papillary carcinomas are reported. Management strategies, including performance of total thyroidectomy, neck dissection, and radioiodine therapy, should be individualized accordingly. Prompt diagnosis and treatment increase survival.

## 4. Conclusion

Thyroid cancer arising from ectopic tissue remains a rare entity. The possibility of an ectopic thyroid cancer in the setting of a normal thyroid gland should be considered as a differential diagnosis in cases of an identified neck mass. Management strategies and intervention should be based on individual risk stratification. Early recognition and differentiation from other identities improve clinical outcomes. This case report demonstrates that a normal thyroid gland does not exclude the presence of thyroid carcinoma in an ectopic tissue.
